# Multiple visible retinal emboli in branch retinal artery occlusion secondary to internal carotid artery occlusion

**DOI:** 10.5935/0004-2749.2022-0206

**Published:** 2024-10-23

**Authors:** Bangtao Yao, Gang Liu, Bin Pang

**Affiliations:** 1 Department of Ophthalmology, Nanjing Lishui People’s Hospital, Zhongda Hospital Lishui Branch, Southeast University, Nanjing, Jiangsu Province, China; 2 Department of Ophthalmology, Xuzhou Third People’s Hospital, Xuzhou Hospital Affiliated to Jiangsu University, Xuzhou, Jiangsu Province, China

A 60-year-old Chinese man presented with 2 day-long painless loss of vision in his right eye. He had systemic diseases, including diabetes mellitus, hypertension, coronary heart disease, lung cancer, and hyperlipidemia, for decades. The best-corrected visual acuity was 20/50 oculus dexter and 20/20 oculus sinister. Fundus examination revealed pale, edematous lesions in the region of the occlusive arteries, as well as multiple visible retinal cholesterol emboli, in the affected arteries. Fundus fluorescein angiography showed delayed fluorescein filling in the early phase (Figure A), and color Doppler ultrasonography showed right internal carotid artery occlusion (Figure B). The patient was immediately treated with sublingual nitroglycerin, massage, salvia miltiorrhiza injection, and oxygenation in the supine position. During the 2-week follow-up, the retinal edema resolved, and partial degradation, peripheral migration, or no movement of the emboli were noted (Figure C). However, the best-corrected visual acuity remained at 20/50.

**Figure 1:**
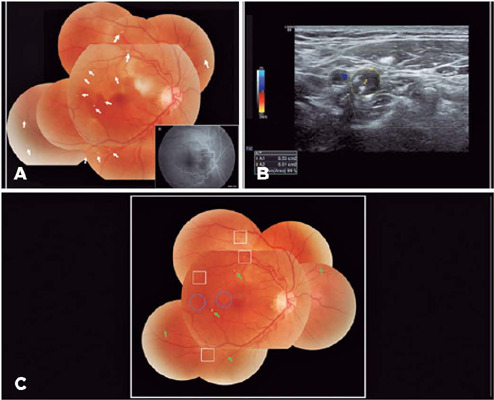
(A) Fundus examination revealed areas of pale, edematous retina in the region of the occlusive artery of her right eye, as well as multiple visible retinal cholesterol emboli. Fundus fluorescein angiography showed delayed fluorescein filling in the early phase. (B) Color Doppler ultrasonography showed internal carotid artery occlusion. (C) During the 2-week follow-up, the retinal edema resolved, and partial degradation (squares), peripheral migration (circles), or no movement (green arrows) of the emboli were noted.

Branch retinal artery occlusion is a severe form of retinal artery occlusive disease, which is characterized by sudden visual reduction and ill-defined retinal infarct^(1)^. The presence of retinal emboli is linked to cardiovascular disease, stroke, and renal disease. The most common cause of retinal cholesterol emboli is severe carotid artery stenosis^(1,2)^.
